# New insight into the management of renal excretion and hyperuricemia: Potential therapeutic strategies with natural bioactive compounds

**DOI:** 10.3389/fphar.2022.1026246

**Published:** 2022-11-22

**Authors:** Bendong Yang, Meiling Xin, Shufei Liang, Xiaoxue Xu, Tianqi Cai, Ling Dong, Chao Wang, Meng Wang, Yuting Cui, Xinhua Song, Jinyue Sun, Wenlong Sun

**Affiliations:** ^1^ School of Life Sciences and Medicine, Shandong University of Technology, Zibo, China; ^2^ Shandong Qingyujiangxing Biotechnology Co., Ltd., Zibo, China; ^3^ Key Laboratory of Novel Food Resources Processing, Ministry of Agriculture and Rural Affairs/Key Laboratory of Agro-Products Processing Technology of Shandong Province/Institute of Agro-Food Science and Technology, Shandong Academy of Agricultural Sciences, Jinan, China

**Keywords:** hyperuricemia, urate transporters, natural products, chronic kidney disease, renal urate extraction

## Abstract

Hyperuricemia is the result of increased production and/or underexcretion of uric acid. Hyperuricemia has been epidemiologically associated with multiple comorbidities, including metabolic syndrome, gout with long-term systemic inflammation, chronic kidney disease, urolithiasis, cardiovascular disease, hypertension, rheumatoid arthritis, dyslipidemia, diabetes/insulin resistance and increased oxidative stress. Dysregulation of xanthine oxidoreductase (XOD), the enzyme that catalyzes uric acid biosynthesis primarily in the liver, and urate transporters that reabsorb urate in the renal proximal tubules (URAT1, GLUT9, OAT4 and OAT10) and secrete urate (ABCG2, OAT1, OAT3, NPT1, and NPT4) in the renal tubules and intestine, is a major cause of hyperuricemia, along with variations in the genes encoding these proteins. The first-line therapeutic drugs used to lower serum uric acid levels include XOD inhibitors that limit uric acid biosynthesis and uricosurics that decrease urate reabsorption in the renal proximal tubules and increase urate excretion into the urine and intestine *via* urate transporters. However, long-term use of high doses of these drugs induces acute kidney disease, chronic kidney disease and liver toxicity. Therefore, there is an urgent need for new nephroprotective drugs with improved safety profiles and tolerance. The current systematic review summarizes the characteristics of major urate transporters, the mechanisms underlying the pathogenesis of hyperuricemia, and the regulation of uric acid biosynthesis and transport. Most importantly, this review highlights the potential mechanisms of action of some naturally occurring bioactive compounds with antihyperuricemic and nephroprotective potential isolated from various medicinal plants.

## Introduction

Uric acid (2,6,8-trioxypurine, C_5_H_4_N_4_O_3_) is a heterocyclic organic compound with a molecular weight of 168.11 Da. It is the end-product of purine (endogenous and exogenous) metabolism in humans and great apes because of loss-of-function mutations during primate evolution in the gene encoding the uricase enzyme, which oxidizes uric acid to produce more soluble allantoin (Wu et al., 1992; So and Thorens, 2010)**.** Due to uricase inactivation, the serum urate level is 7- to 8-fold higher in humans (≈240–360 μM) than in other mammals (≈30–50 μM in mice) (So and Thorens, 2010). Considering the pKa value of uric acid (5.75) that exists as soluble urate at physiological pH (7.4), more uric acid than urate is present in the urine (pH 5–6), and a pH less than 7.4 in the tissue microenvironment favors urate crystal formation. In humans, the serum urate concentration is approximately 10 times higher than that of ascorbic acid, showing that urate is a potent radical scavenger in plasma. Perhaps 7- to 8-fold higher serum urate levels might have selectively protected the neurons of hominids during evolution from the detrimental effects of reactive oxygen species (ROS) and allowed them to become the most intelligent species on Earth. In addition, elevated serum urate levels have been shown to reduce the risk of neurodegenerative diseases, specifically Alzheimer’s disease (Ye et al., 2016), Parkinson’s disease (Schwarzschild et al., 2008; Ascherio et al., 2009), and multiple sclerosis (Liu et al., 2012). Recently, integral membrane protein 2B (ITM2B) has been shown to be a potential regulatory link between urate homeostasis and neurodegenerative disorders (Mandal and Mount, 2019). Elevated serum urate levels may have given hominids a selective advantage during evolution. Taken together, these results suggest a potential role of soluble urate in maintaining memory and intelligence.

Approximately two-thirds of serum uric acid in humans is produced endogenously, while the remaining third comes from dietary purines (Schlesinger 2005). Under physiological concentrations, uric acid in its soluble form as urate acts as a protective powerful antioxidant, demonstrating the ability to scavenge ROS such as superoxide, hydroxyl radicals, and singlet oxygen (Ames et al., 1981; Davies et al., 1986), which is closely comparable to the scavenging ability of vitamin C (Ames et al., 1981). However, under conditions of poor solubility and a high uric acid concentration in crude urine, urate crystal deposition occurs in the renal tubular lumens and ureters, which contributes to obstructive nephropathy (Moe et al., 2002). The urate crystals adhere to the surfaces of renal epithelial cells and induce an acute inflammatory response (Koka et al., 2000). In addition to inducing kidney stone formation, such effects can reduce the glomerular filtration rate (Spencer et al., 1976). Urate can also act as a pro-oxidant inside cells, as it can induce the activity of NADPH oxidases, resulting in mitochondrial alterations and endothelial dysfunction (Sánchez-Lozada et al., 2012).

Hyperuricemia is a common finding in patients with metabolic syndrome. Clinically, the prevalence of hyperuricemia is much higher than that of hypouricemia. Hyperuricemia in humans is defined by the serum urate level >7.0 mg/dl among men and >5.7 mg/dl among women**.** Statistically, the morbidity due to hyperuricemia and gout is higher in men than in women, probably because of the influence of sex hormones. With increasing age after menopause, the discrepancy in prevalence is reduced between men and women (Chen-Xu et al., 2019). The prevalence of hyperuricemia increased from 10.5% to 16.6% in Caucasian or Australian representative populations from 2011 to 2020 (Pathmanathan et al., 2021). In Ireland, the prevalence increased from 19.7% to 25.0% among men and from 20.5% to 24.1% among women between 2006 and 2014 (Kumar A U et al., 2018). The pooled prevalence of hyperuricemia was 19.4% in men and 7.9% in women from 2000 to 2014 and was high in mainland China (Liu et al., 2015). Long-term hyperuricemia can induce renal mitochondrial dysfunction associated with oxidative stress in the renal cortex as well as tubular damage (Cristóbal-García et al., 2015). Moreover, it can lead to urate crystal formation that causes gout (a type of inflammatory arthritis), leading to joint damage, loss of motion and an acute inflammatory response (Koka et al., 2000). Hyperuricemia is the causative risk factor for gout (Choi et al., 2005) and increases the risks for chronic kidney disease (CKD) (Johnson et al., 2018), cardiovascular disease (Feig et al., 2008; Gaffo et al., 2009), hypertension (Feig and Johnson, 2003), insulin resistance (Facchini et al., 1991; Yoo et al., 2005), and diabetic kidney disease (Kim et al., 2012; Qu et al., 2022). Currently, it is recognized that hyperuricemia alone is not sufficient to cause gout and that other factors also play roles in urate crystal formation. It is also acknowledged that lowering serum urate levels is an effective strategy to prevent gout attacks (Shoji et al., 2004).

The normal serum urate level in humans is the result of balance among biosynthesis of uric acid primarily in the liver, reabsorption of urate in renal proximal tubules and secretion in the renal tubules and intestine (Mandal and Mount, 2015). Dysregulation of xanthine oxidoreductase (XOD), the enzyme that catalyzes the endogenous production of uric acid primarily in the liver, and urate transporters that reabsorb urate in renal proximal tubules and secrete urate in renal tubules and the intestine, is the major cause of hyperuricemia, along with variability in the genes encoding these proteins. Notably, most urate is filtered freely in the kidney, with approximately 90% of the urate from glomerular filtrate being reabsorbed *via* urate transporters in the proximal tubules (Alexandru and Moe, 2012). Approximately 70% of the total serum urate in the human body is excreted *via* the kidney, and the rest is excreted *via* intestinal and biliary secretion (Schlesinger 2005). Ultimately, after urate reabsorption, only 3–10% of the filtered urate is eliminated in the urine (Taniguchi and Kamatani, 2008). Abnormalities in urate metabolism and decreased urate metabolite in the kidney are major inducers of hyperuricemia and gout development (Terkeltaub 2003; Liu et al., 2015). Genome-wide association studies have also found associations between polymorphisms in urate transporters and the risk for hyperuricemia/gout (Köttgen et al., 2013; Phipps-Green et al., 2016; Tin et al., 2019). In this review, advances in urate excretion and possible therapeutic herbal extracts are discussed to provide novel insights regarding the development and treatment of hyperuricemia.

### Relationship between hyperuricemia and CKD

Long-term hyperuricemia is considered an independent risk factor for the occurrence and progression of CKD (Jalal et al., 2013; Su et al., 2020). The prevalence of hyperuricemia and CKD has been steadily increasing (Levey et al., 2007; Jalal et al., 2013). Patients with long-term hyperuricemia have poor quality of life and high mortality rates. Because of advances in research, CKD can now be detected using simple laboratory tests, and there are treatments to prevent or delay abnormal kidney function, slow the progression of kidney disease, and reduce the risk of CKD. However, the debate regarding whether hyperuricemia plays a causal role in the progression of CKD or is simply a marker of renal dysfunction continues. The causal relationship between hyperuricemia and CKD remains controversial, and the pathophysiological mechanisms of hyperuricemia-induced renal injury are not entirely clear. Serum urate is often elevated in subjects with CKD but is not always associated with the development and progression of CKD. The causal role of hyperuricemia in the progression of CKD has not been fully established. Currently, there is no clear cutoff serum urate level associated with the risk for kidney damage, and there is not sufficient evidence to recommend the widespread use of uric acid-lowering therapy to prevent or slow the progression of CKD. Elevated urate levels in PO-induced (PO, potassium oxonate; a uricase inhibitor) hyperuricemic rats have been shown to cause intrarenal oxidative stress, increased NOX-4 and angiotensin II expression, increased juxtaglomerular renin and decreased nitric oxide bioavailability, renal vasoconstriction, glomerular hypertrophy, glomerulosclerosis and afferent arteriolopathy (Mazzali et al., 2001; Kang et al., 2002; Nakagawa et al., 2003; Sánchez-Lozada et al., 2005; Sánchez-Lozada et al., 2008). Furthermore, treatment with allopurinol partially prevents cortical vasoconstriction and fully prevents arteriolopathy and glomerular hypertension (Nakagawa et al., 2003; Sánchez-Lozada et al., 2005).

Excessive serum urate causes hyperuricemic nephropathy, which is characterized by inflammatory infiltration of macrophages, neutrophils and lymphocytes and tubulointerstitial fibrosis (Lai and Zhou, 2013; Pan et al., 2021a). Recent findings suggest that asymptomatic hyperuricemia has no effect on CKD progression unless urate crystallizes in the kidney (Sellmayr et al., 2020). However, when experimental animals with CKD are made hyperuricemic, renal disease progresses rapidly despite an absence of crystals in the kidney. This experimental results link hyperuricemia with the progression of CKD as in most mammals, uric acid levels are relatively low (compared to those in humans) because of the presence of liver uricase, which degrades uric acid to 5-hydroxyisourate and eventually to soluble allantoin. All humans are essentially “uricase knockouts,” exhibiting elevations in serum urate levels that can be treated. Reducing urate levels with losartan may slow renal disease (Miao et al., 2011) and reduce cardiovascular events (Smink et al., 2012). The renoprotective drug febuxostat (a xanthine oxidase inhibitor) does not alleviate the decline in kidney function in patients with stage 3 CKD and asymptomatic hyperuricemia (Kimura et al., 2018). Evidence obtained from basic research suggests that hyperuricemia plays a pathogenic role in the development of CKD and cardiovascular disease by inducing inflammation, endothelial dysfunction, proliferation of vascular smooth muscle cells, and activation of the renin–angiotensin system (Saito et al., 1978; Mazzali et al., 2001; Perlstein et al., 2004). In human first trimester uterine trophoblast cell lines, monosodium urate (MSU) crystals have been shown to induce inflammatory cytokine production in response to activation of the NOD-like receptor superfamily pyrin domain containing 3 (NLRP3) inflammasome (Mulla et al., 2011; Mulla et al., 2013). CKD is most often associated with obesity and metabolic syndrome (Copur et al., 2022), and there is insufficient evidence to suggest that uric acid-lowering therapy can prevent CKD progression.

### Relationship between hyperuricemia and insulin resistance

Serum urate is elevated in metabolic syndrome and diabetes (Choi et al., 2007; Choi and Ford, 2007; Copur et al., 2022) as a consequence of insulin resistance and insulin-mediated reductions in urinary urate excretion (Quiñones Galvan et al., 1995). There is a positive relationship between serum insulin and elevated serum urate levels in healthy individuals and people with diabetes (Mandal et al., 2021). Insulin resistance also leads to impaired urate excretion at a low urinary pH, contributing to the formation of urate stones (Spatola et al., 2017). Genetic variation in insulin signaling pathways is also associated with variations in serum urate levels (Köttgen et al., 2013; Phipps-Green et al., 2016; Tin et al., 2019). These genetic data are consistent with a role of insulin in controlling serum urate levels (Mandal et al., 2021). In an oocyte expression system and transfected cells overexpressing individual urate transporters, insulin was recently suggested to activate both “reabsorptive” urate transporters (GLUT9 and OAT10) and “secretory” urate transporters (OAT1, OAT3, NPT1, and ABCG2) *via* phosphoinositide 3-kinase/protein kinase (PI3K/AKT) and mitogen-activated protein kinase/extracellular signal-regulated kinase signaling pathways (Mandal et al., 2021).

## Various urate transporters involved in renal excretion

Well-characterized urate transporters that are involved in reabsorption of urate from the glomerular filtrate in the human renal proximal tubules include human urate transporter 1 (URAT1, encoded by the *SLC22A12* gene), organic anion transporters (OAT10/ORCTL3, encoded by the *SLC22A13* gene, and OAT4, encoded by the *SLC22A11* gene), and glucose transporter member 9 (GLUT9, encoded by the *SLC2A9* gene). The urate transporters that are involved in secretion of urate into the urine from serum include OAT1 (encoded by the *SLC22A6* gene), OAT2 (encoded by the *SLC22A7* gene), OAT3 (encoded by the *SLC22A8* gene), ATP-binding cassette, subfamily G, member 2 (ABCG2, encoded by the *ABCG2* gene), ABCC4 (encoded by the *ABCC4* gene), sodium-dependent phosphate transporters types (NPT1, encoded by the *SLC17A1* gene) and NPT4 (encoded by the *SLC17A3* gene) (Mandal and Mount, 2015; Mandal et al., 2017). Urate reabsorption in the proximal tubule involves the coordinated activity of several transporters. Sodium-dependent reabsorption of organic monocarboxylates by the apical Na^+^-dependent monocarboxylate transporters SMCT1 (encoded by the *SLC5A8* gene) and SMCT2 (encoded by the *SLC5A12* gene) (Coady et al., 2004; Srinivas et al., 2005; Mandal et al., 2017) increases the intracellular concentrations of monocarboxylate anions that can then be exchanged with luminal urate *via* the urate–anion exchangers URAT1 and OAT10.

### URAT1 (SLC22A12)

**FIGURE 1:**
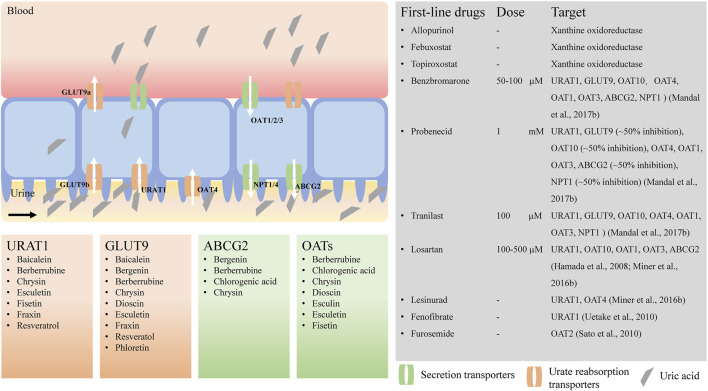
Urate transporters and potential natural products for hyperuricemia treatment. URAT1, urate transporter 1; GLUT9, glucose transporter member 9; ABCG2, ATP-binding cassette transporter, subfamily G, member 2; OATs, organic anion transporters; NPTs, sodium-dependent phosphate cotransporter types. The activities of urate transporters were inhibited by benzbromarone (Mandal et al., 2017), probenecid (Mandal et al., 2017), tranilast (Mandal et al., 2017), losartan (Hamada et al., 2008; Miner et al., 2016b), lesinurad (Miner et al., 2016b), fenofibrate (Uetake et al., 2010), furosemide (Sato et al., 2010), respectively.

URAT1 is expressed in the apical membrane of the proximal tubules in the kidney and has been regarded as the dominant apical urate/anion exchanger in humans mediating urate reabsorption (Figure 1). URAT1 transports urate in exchange for intracellular nicotinate and pyrazinoate, but not lactate (Mandal et al., 2017). In an oocyte expression system, the urate/anion exchange activity of URAT1 has been found to be independent of Na^+^ ions, but complete removal of Cl^−^ ions in the extracellular medium increases the urate uptake activity of URAT1 3- to 4-fold. URAT1 is also transported in exchange for pyrazinoate. Uricosuric drugs such as benzbromarone, probenecid, tranilast (Mandal et al., 2017), lesinurad (Miner et al., 2016b; Zhao et al., 2020), fenofibrate (Uetake et al., 2010), and losartan, an angiotensin II receptor antagonist (Nakashima et al., 1992), are potent inhibitors of URAT1.

Loss-of-function mutations in the *SLC22A12* gene are associated with hypouricemia (Dinour et al., 2011). Hypouricemia has been previously shown to be associated with common variants associated with several single nucleotide polymorphisms (SNPs), including rs12800450 (G65W), rs121907896 (R90H), rs121907892 (W258X), rs139316841 (W277R), rs749900943 (R349W), rs147647315 (R434H), rs201423508 (T450I) and rs747742344 (E458K) (Ichida et al., 2004; Tin et al., 2011; Sakiyama et al., 2016). The identification of missense mutations (T217M, E298D, G269A, R406C, G412A, G444R, G490A, A1145T, and T1289C) in patients with hypouricemia validates URAT1 as the primary reabsorption urate transporter (Enomoto et al., 2002; Ichida et al., 2004; Dinour et al., 2011). In an *SLC22A12*-knockout mouse model, fractional urate excretion was significantly greater than that in wild-type mice, confirming the role of URAT1 in urate reabsorption (Hosoyamada et al., 2010). Based on this information, regulation of the URAT1 expression level and its activity is an effective strategy for maintaining a suitable serum urate concentration. Benzbromarone is a potent uricosuric agent that has been used in the treatment of gout for over 30 years. It functions by increasing urate excretion in human kidney proximal tubules through effective inhibition of the dominant apical (luminal) urate exchanger URAT1 at low doses (Enomoto et al., 2002; Mandal et al., 2017). This blockade reduces urate reabsorption, increasing urate elimination *via* the urine (Enomoto et al., 2002). Benzbromarone was withdrawn from the market by Sanofi-Synthelabo in 2003 after reports of severe hepatotoxicity (Jansen et al., 2004). Aggravation of hepatic steatosis in obese individuals and gastrointestinal problems associated with benzbromarone treatment limit the clinical use of this drug but warrant further affirmation *in vivo* (Heel et al., 1977; Sun et al., 2018). Recently, many studies have indicated that SNPs in the *SLC12A22* gene are strongly associated with hyperuricemia and gout. In an analysis of the Korean Cancer Prevention Study-II (KCPS-II) cohort, sequencing of URAT1 in 68 male Korean subjects revealed that the pattern most strongly associated with hyperuricemia included a common variant consisting of rs7929627 (IVS7-103A/G, noncoding variants), rs75786299 (IVS3+11A/G, noncoding variants) and rs3825017 (N82N, coding variant). Moreover, rs11602903 (788A/T, promoter variants) and rs121907892 (W258X, coding variant) were negatively correlated with hyperuricemia (Cho et al., 2015). In addition, other SNPs, including rs3825017 (N82N), rs3825016 (C/T), and rs11231825(H142H), are linked with the risk of hyperuricemia and gout (Li et al., 2014; Pavelcova et al., 2020).

### GLUT9 (*SLC2A9*)

GLUT9 is a voltage-driven high-capacity urate transporter that is mainly expressed in the proximal tubules of the human kidney, the liver, and the intestine (Figure 1) (Anzai et al., 2008; Döring et al., 2008; Vitart et al., 2008; Wallace et al., 2008). It is the sole transporter that transports reabsorbed urate from the proximal tubular epithelium to the blood. Loss-of-function mutations in GLUT9 have been identified in familial hypouricemia, and SNPs are associated with reduced serum urate, indicating that GLUT9 is a major determinant of serum urate levels (Mandal and Mount, 2015). GLUT9 exists in two isoforms, GLUT9a and GLUT9b, which differ in their amino-terminal cytoplasmic domains; GLUT9a is located in the basolateral membrane, and GLUT9b is located in the apical membrane of the proximal tubules in human kidney (Augustin et al., 2004; Kimura et al., 2014). However, in mice, Glut9a is expressed in the proximal convoluted and straight tubules, and Glut9b is expressed in distal convoluted tubules and connecting tubules (Bibert et al., 2009). Thus, mouse Glut9 in the kidney differs from human GLUT9. Human GLUT9a is 540 amino acids in length and is encoded by 12 exons, whereas GLUT9b is 511 amino acids in length and is encoded by 13 exons of the splice variants of RNA of the *SLC2A9* gene. In both humans and mice, GLUT9b is expressed only in the liver and kidney, whereas GLUT9a is present in many more tissues, such as the liver, kidney, intestine, leukocytes, and chondrocytes (Augustin et al., 2004; Keembiyehetty et al., 2006; Kimura et al., 2014).

Although GLUT9 was initially reported to mediate glucose/fructose transport (Augustin et al., 2004; Doblado and Moley, 2009), it was later confirmed to be a high-capacity robust urate transporter without any detectable glucose/fructose transport activity (Caulfield et al., 2008; Vitart et al., 2008; Mandal et al., 2017; Mandal et al., 2021). Furthermore, *SLC2A9*-knockout mice exhibit increased serum urate levels with impaired enterocyte urate transport kinetics. *SLC2A9* deficiency in mice can induce the occurrence of early-onset metabolic syndrome, suggesting a role of *SLC2A9* in regulating enterocyte urate clearance (DeBosch et al., 2014). Importantly, uricosuric drugs such as benzbromarone, probenecid, and tranilast (Mandal et al., 2017), but not losartan or lesinurad, are potent inhibitors of GLUT9 (Miner et al., 2016a). In a oocyte expression system, urate uptake activity was found to be almost completely inhibited by 100 µM benzbromarone (Mandal et al., 2017). The uptake of urate mediated by GLUT9 is inhibited by benzbromarone (100 µM) and losartan (1 mM) by approximately 90 and 50%, respectively (Bibert et al., 2009). Moreover, electrophysiological measurements suggest that urate transport by mouse GLUT9 is electrogenic and voltage-dependent but independent of Na^+^ and Cl^−^ transmembrane gradients (Bibert et al., 2009). However, in the oocyte expression system, complete removal of Cl^−^ ions in the extracellular medium increased the urate uptake activity of GLUT9 3- to 4-fold. Similarly, complete replacement of Na^+^ ions by K^+^ ions in the extracellular medium increased the urate uptake activity of human GLUT9 4- to 5-fold (Mandal et al., 2017).

### ABCG2 (ABCG2)

ABCG2 protein, first identified as a multidrug resistance protein (Doyle et al., 1998) that transports a wide range of structurally and functionally diverse chemotherapeutics, has been characterized as a high-capacity urate secretion transporter (Matsuo et al., 2009; Woodward et al., 2009; Nakayama et al., 2011). ABCG2 is located on chromosome 4q, as identified by genome-wide association studies related to hyperuricemia and gout (Nakayama et al., 2011). It is expressed in the apical membrane of the human kidney proximal tubules (Figure 1) (Huls et al., 2008; Woodward et al., 2009), the intestine and the liver (Nakayama et al., 2011).

In white, African and Asian populations, the SNP rs2231142 in exon 5 of the ABCG2 gene, which generally causes the Gln141Lys amino acid substitution, is the strongest link between ABCG2 and gout/hyperuricemia (Dehghan et al., 2008). Functional studies have shown that the Q141K substitution causes an approximately 53% reduction in ABCG2-mediated urate export activity compared with that of the wild-type protein (Woodward et al., 2009). Approximately one-third of uric acid is excreted from the intestines in humans (Matsuo et al., 2014). Thus, ABCG2 plays a crucial role as an essential renal and intestinal urate exporter, as its dysfunction is associated with abnormal serum uric acid levels and gout/hyperuricemia risk. In an oocyte expression system, benzbromarone and probenecid were shown to inhibit ABCG2 urate export activity (Mandal et al., 2017; Fujita et al., 2019), but lesinurad was not (Miner et al., 2016a).

### OATs (SLC22A)

OATs, encoded by *SLC22* family of genes, are expressed in the barrier epithelia of the major excretory organs of the body, the kidney and the liver (Figure 1). These transporters play important roles in renal drug elimination, as they interact with endogenous metabolic end products such as urate and a multitude of widely used drugs, including antibiotics, antihypertensives, antivirals, anti-inflammatory drugs, diuretics and uricosurics (Rizwan and Burckhardt, 2007). OATs also play important roles in both the renal secretion and reabsorption of urate. Five of the characterized OATs are expressed in the renal proximal tubules: OAT1 (encoded by the *SLC22A6* gene), OAT2 (encoded by the *SLC22A7* gene) and OAT3 (encoded by the *SLC22A8* gene) are located in the basolateral membrane, whereas OAT4 (encoded by the *SLC22A11* gene) and OAT10/ORCTL3 (encoded by the *SLC22A13* gene) are located in the apical membrane of the renal proximal tubules (Eraly et al., 2008). The basolateral urate transporters OAT1, OAT2 and OAT3 function in urate secretion (Eraly et al., 2008), transporting urate from blood into proximal tubular cells for secretion at the apical membrane (Mandal et al., 2017). OAT1 and OAT3 are Na^+^-independent and exchange urate with divalent anions such as α-ketoglutarate (Aslamkhan et al., 2003; Bakhiya et al., 2003; Sweet et al., 2003), indicating that basolateral entry of urate from the blood into the proximal tubular epithelium is driven by intracellular α-KG during urate secretion. In an oocyte expression system, the urate transport activity of murine OAT3 was found to be cis-inhibited by extracellular salicylate, nicotinate or pyrazinoate anion (Mandal et al., 2017). OAT2 is chloride-dependent and has more restricted substrate specificity than OAT1 and OAT3 (Sato et al., 2010). The urate transport activity of OAT2 is cis-inhibited by the antiuricosuric agents pyrazinecarboxylic acid and nicotinate (Sato et al., 2010), and OAT3 is cis-inhibited by pyrazinecarboxylic acid, nicotinate and salicylate (Mandal et al., 2017). Human OAT4 transports urate in exchange for divalent organic dicarboxylate ions (Hagos et al., 2007; Mandal et al., 2017). OAT10 is a urate transporter and high-affinity urate/nicotinate exchanger dependent on Cl^−^ ions (Bahn et al., 2008; Mandal et al., 2017). OAT10 and URAT1 share functional similarities, as both of them transport urate and nicotinate and as the urate transport activity of both is trans-stimulated by intracellular nicotinate or pyrazine carboxylate (Mandal et al., 2017). Oat1-and Oat3-null mice exhibit decreased secretion of urate rather than reabsorption (Eraly et al., 2008). Benzbromarone, probenecid and tranilast were shown to inhibit the urate transport activity of OAT10, OAT4, OAT1, and OAT3 in an oocyte expression system (Mandal et al., 2017). Benzbromarone, probenecid, losartan, telmisartan, hydrochlorothiazide, and furosemide have been shown to inhibit the urate transport activity of OAT2 (Sato et al., 2010). In addition, lesinurad inhibits OAT4 but does not inhibit OAT1 or OAT3 in the clinical setting (Sato et al., 2010).

### NPTs (SLC17A)

NPT1 and NPT4, encoded by *SLC17A1* and *SLC17A3* genes, respectively, are located at the apical membrane of the proximal tubule and mediate net tubular urate secretion (Figure 1). Genome-wide association studies have identified a region in chromosome 6p23-p21.3, where *SLC17A1* and *SLC17A3* are located, that is associated with serum urate concentrations (Kolz et al., 2009). NPT1 is the first identified member of the *SLC17A* phosphate transporter family. Human NPT1 transports organic anions such as urate, p-aminohippurate and acetylsalicylate (aspirin), and salicylate in a voltage-driven and Cl^−^-dependent manner (Reimer and Edwards, 2004; Iharada et al., 2010). The identification of a common gain-of-function variant, rs1165196 (T806C), in Japanese patients with significantly decreased risk of renal underexcretion gout enhanced understanding of the physiological role of NPT1 as a renal urate exporter (Chiba et al., 2015). Benzbromarone, probenecid and tranilast have been shown to inhibit the urate transport activity of NPT1 in an oocyte expression system (Mandal et al., 2017). NPT4 is also a voltage-dependent organic anion transporter, similar to porcine OAT1/3, with lower affinity urate transport (Jutabha et al., 2011). Two thiazide drugs, chlorothiazide-2 and trichlormethiazide-3, and two loop diuretics, bumetanide-4 and furosemide-5, have been shown to inhibit the urate transport activity of NPT4 in an oocyte expression system (Jutabha et al., 2011), which suggests the involvement of NPT4 in diuretic-induced hyperuricemia. Two SNPs, rs1165205 within intron 1 of *SLC17A3* and rs116205 identified in the *SLC17A3* gene, correlate well with the changes in serum urate concentration (Riches et al., 2009). The *in vivo* role of NPT4 is supported by the presence of missense mutations (N68H and F304S) in *SLC17A3* in underexcretion-type hyperuricemia patients (Jutabha et al., 2010).

## Management of hyperuricemia by herbal extracts and natural bioactive compounds

The field of traditional Chinese medicine (TCM) is valued for its holistic view of the human body. TCM provides a massive amount of information on natural products and disease phenotypes observable as clinical symptoms that are crucial for clinical diagnosis and treatment (Table 1). Various aqueous or ethanolic herbal extracts used in TCM have been demonstrated to be beneficial in various disease conditions. The 70% ethanol extract (comprising polyphenols and flavonoids) of *Eurycoma longifolia*, a tropical medicinal plant, has been reported to significantly reduce serum urate levels by downregulating the protein expression levels of Urat1 and Glut9 in rats with PO-induced hyperuricemia and an adenine-/PO-induced hyperuricemia mouse model (Bao et al., 2019). The macroporous resin extract of *Dendrobium officinale* leaves has been reported to reduce serum urate levels in rats with fructose- and PO-induced hyperuricemia by inhibiting XOD activity and regulating the expression of Abcg2, Urat1, and Glut9 (Wang et al., 2022). Sunflower calathide aqueous extract has been shown to reduce serum urate levels to a degree comparable to that mediated by allopurinol and benzbromarone in rats with yeast extract-induced hyperuricemia with renal injury (Dai et al., 2021). Sunflower calathide aqueous extract has also been shown to downregulate the cytokines COX-2, PGE2, NO, and IFN-γ in lipopolysaccharide (LPS)-treated RAW264.7 cells (Dai et al., 2021). Ethanol extract of the bark of *Liriodendron chinense* (Hemsl.) Sarg has been shown to reduce serum urate in adenine and PO-induced hyperuricemia mice with nephropathy by suppressing the activation of nuclear factor-kappa B (NF-κB) and the Janus kinase/signal transducer and activator of transcription 3 (JAK/STAT3) signaling pathway, reducing inflammatory factor infiltration and urate accumulation in the kidney (Pan et al., 2021a). A polysaccharide (molecular weight, 46.56 kDa) obtained from the green algae *Enteromorpha prolifera*, comprising rhamnose, glucuronic acid, galactose, arabinose, and xylose at a molar ratio of 20.45:12.74:10.99:5.84:1.95, has been shown to significantly reduce serum urate, serum XOD and hepatic XOD; upregulate the mRNA and protein expression of urate secretion transporters Abcg2, Oat1, and Npt1; and downregulate the urate reabsorption transporter, maintaining the stability of the intestinal flora in mice with hyperuricemia (Li X. et al., 2021). Fufang Zhenzhu Tiaozhi capsule prevents renal injury, inflammation and fibrosis in mice with hypoxanthine- and PO-induced hyperuricemia by promoting urate excretion and inhibiting the PI3K/AKT/NF-κB signaling pathway (Li et al., 2022). The Third National Health and Nutrition Examination Survey (1988–1994), using data from 14,758 men and women aged ≥20 years, showed that coffee consumption is associated with reduced serum urate levels (Choi and Curhan, 2007). Dioscin, a spirostane glycoside with anti-inflammatory and antiallergic properties found in the rhizome of *Dioscorea spongiosa*, has been shown to significantly reduce serum urate by downregulating Glut9 and upregulating Oat1 in rats with PO-induced hyperuricemia alone and in mice with adenine-/PO-induced hyperuricemia (Tao et al., 2018; Zhang et al., 2018; Li J. et al., 2021). Theobromine, a natural dimethylxanthine present in high amounts in cocoa, may be clinically useful in the treatment of nephrolithiasis, as it inhibits nucleation and urate crystal growth (Grases et al., 2014). Curcumin, a hydrophobic polyphenol extracted from the rhizome of *Curcuma longa*, has been shown to significantly reduce serum urate in PO-induced hyperuricemia rats modulating the gut microbiota, fortifying the intestinal barrier, attenuating metabolic endotoxemia, and consequently protecting renal function (Xu et al., 2021).

**TABLE 1:** Summary of the effects and potential mechanisms of candidates for hyperuricemia treatment. PO, potassium oxonate; LPS, lipopolysaccharide; URAT1, human urate transporter 1; GLUT9, glucose transporter member 9; ABCG2, ATP-binding cassette transporter, subfamily G, member 2; OAT, organic anion transporter; NPT, sodium-dependent phosphate cotransporter type; NLRP3, NOD-like receptor family pyrin domain-containing 3; IL-6, interleukin-6; IL-1β, interleukin-1β; NF-κB, nuclear factor-kappa B; TGF-β, transforming growth factor-beta; PI3K/AKT, phosphoinositide 3-kinase/protein kinase; JAK/STAT3, Janus kinase/signal transducer and activator of transcription 3, TLR4, Toll-like receptor 4; XOD, xanthine oxidoreductase.

Bioactive compound	Natural resources	Effects	Potential mechanisms	Models	References
Anthocyanin s (flavonoids)	Korean black beans (also found in blue, purple, and red colored fruits, flowers and leaves)	Significantly reduces serum urate levels, antioxidant, neuroprotective	Inhibits XOD, inhibits activation of PI3K/Akt/GSK3β	Mice with yeast extract-induced hyperuricemia	(Ali et al., 2018; Qian et al., 2019)
Chrysin (a flavonoid)	Honey, propolis, mushrooms	Effectively reduces serum urate levels, anti-inflammatory, antioxidant	Inhibits XOD, downregulates URAT1 and GLUT9 expression, upregulates OAT1 and ABCG2 expression, inhibits activation of the PI3K/Akt/mTOR signaling pathway and the NLRP3 inflammasome	High-fructose corn syrup-fed hyperuricemia rats, RAW264.7 cells	(Chen et al., 2021a; Cai et al., 2021; Chang et al., 2021)
Bergenin (a type of polyphenolic compound)	Medicinal plants like *Bergenia crassifolia, Corylopsis spicata, Rodgersia sambucifolia*	Significantly reduces serum urate levels, antiulcerogenic, anti-inflammatory, wound-healing	Induces Abcg2 expression, suppresses Glut9 expression, inhibits nuclear translocation of p53, inhibits activation of PI3K/Akt signaling pathway, decreases the serum levels of IL-6, IL-1β, and TNF-α	Model fed daily with diet mixed with 25% yeast polysaccharide, mice with PO-induced hyperuricemia, HK-2 cells, Caco-2 cells, RAW264.7 cells	Chen et al. (2020)
Baicalein (a bioactive flavonoid)	Roots of the Chinese herbs *Scutellaria baicalensis* and *Scutellaria lateriflora*	Significantly reduces serum urate levels, antioxidant, anti-inflammatory, antihypertensive, anticancer	Inhibits the activity of Glut9 and Urat1, inhibits XOD activity, inhibits activation of the PI3K/AKT/NF-κB pathway	Mice with PO-induced hyperuricemia, human A549 lung adenocarcinoma cells	(Yu et al., 2017; Chen et al., 2021b)
Berberrubine (an isoquinoline alkaloid)	Phellodendri Chinensis Cortex, *Coptis chinensis* Franch and *Phellodendron chinense* Schneid	Significantly reduces serum urate levels, anti-gout	Inhibits hepatic XOD activity, downregulates the GLUT9 and URAT1 expression, upregulates OAT1/3 and ABCG2 expression, reduces inflammatory mediator (IL-1β, IL-6, and TNF-α) levels, suppresses the JAK2/STAT3 signaling pathway	Mice with PO- and hypoxanthine-induced hyperuricemia	(Lin et al., 2021; Cheng et al., 2022a)
Pectolinarigenin (a flavonoid)	*Crisium setidens, Cirsium chanroenicum* and citrus fruits	Significantly reduces serum urate levels, alleviates inflammation and fibrosis	Inhibits the TGF-β expression and phosphorylation of the transcription factors Smad3 and Stat3, LPS-induced NF-κB activation, and synthesis of iNOS, COX-2, IL-6, IL-1β, and TNF-α	Mice with adenine- and PO -induced hyperuricemic nephropathy, uric acid-treated mouse kidney epithelial cells, RAW264.7 and THP1 cell lines	(Ren et al., 2021a; Feng et al., 2022)
Fisetin (a flavonol)	Widely dispersed in fruits (apples, grapes, strawberries), vegetables and nuts	Reduces serum urate, anti-inflammatory, attenuates hyperuricemia-induced kidney injury, improves renal function, and inhibits tumor growth, osteoarthritis, and rheumatoid arthritis	Modulates/restores the expression of Urat1, Oat1, Oat3 and Abcg2; reduces the levels of inflammatory mediators (IL-6, TNF-α); regulates activation of the JAK/STAT3 and TGF-β signaling pathways; inhibits activation of the FGFR1/TLR4/NLRP3 inflammasome pathway	Mice with adenine- and PO-induced hyperuricemic nephropathy, uric acid-stimulated mouse kidney tubular epithelial cells	(Ren et al., 2021b; Huang et al., 2021)
Phloretin (a type of natural phenol)	Leaves of apple and apricot trees	Reduces serum urate levels, antioxidant, anti-inflammatory	Inhibits urate reabsorption mediated by Glut9, suppresses the NLRP3 signaling pathway, inhibits uric acid-induced activation of the ERK/NF-κB pathway	Mice with PO-/adenine-induced hyperuricemia, human umbilical vein endothelial cells	(Liu et al., 2017; Cui et al., 2020)
Resveratrol (a polyphenol)	Grapes, veratrum and other plants	Reduces serum urate levels, anti-inflammatory, antioxidant, antidiabetic, antiarthritic, anticancer, neuroprotective	Downregulates the expression levels of Glut9 and Urat1; reduces the renal concentrations of IL-6, IL-18, IL-1β and TNF-α; inhibits the Nlrp3 inflammasome and TLR4/MyD88/NF-kB, Nrf2 and NF-κB signaling pathways	Mice with high-fat diet-induced insulin resistance, Sprague‒Dawley rat model of sepsis peritonitis, duck ileitis caused by LPS	(Shang et al., 2019; Yang et al., 2021; Zhang et al., 2021)
Chlorogenic acid (a polyphenol)	Medicinal herbs, apples, artichokes, carrots, eggplants, grapes, cherries, honeysuckle, sunflower	Reduces serum urate levels; antioxidant and anti-inflammatory activity; alleviates kidney fibrosis, liver fibrosis, metabolic syndrome, acute kidney injury, and diabetic nephropathy	Downregulates the mRNA expression of secretory urate transporters, inhibits the PI3K/AKT/mTOR and the NF-κB signaling pathways, reduces the mRNA expression of interleukin IL-1β, tumor necrosis factor TNF-α, Nlrp3, and caspase-1, and the TLR4/MyD88/NF-κB signaling pathway in kidney	Mice and rats with PO/hypoxanthine-induced hyperuricemia	(Ye et al., 2017; Zhou et al., 2021)
Esculetin (a natural dihydroxy coumarin)	*Fraxinus rhynchophylla* bark and chicory skin	Significantly reduces serum urate, antioxidant, anti-inflammatory, antiapoptotic, anticancer, antidiabetic, neuroprotective	Inhibits XOD in liver; modulates urate transporters in the kidney; upregulates OAT1, IL-6, IL-1β, and TNF-α; suppresses the expression of inducible nitric oxide synthase and the cyclooxygenase-2 protein by blocking the NF-κB pathway	Mice with PO-induced hyperuricemic nephropathy, mice with unilateral ureteral occlusion, RAW264.7 macrophages, HepG2 and NRK-52E cells	(Hong et al., 2014; Zhu et al., 2016; Wang et al., 2020; Zhang et al., 2022)
Esculin (a coumarin derivative)	*Fraxinus rhynchophylla* bark and chicory skin	Significantly reduces serum urate, anti-inflammatory, antiarthritic and anticancer	Upregulates Oat1	PO-induced hyperuricemic nephropathy	(Li et al., 2011; Zhou et al., 2018)
Fraxin (a coumarin derivative)	*Fraxinus rhynchophylla* bark and chicory skin	Significantly reduces serum urate, anti-inflammatory, antiarthritic and anticancer	Inhibits Glut9 and Urat1	Mice with PO-induced hyperuricemic nephropathy	(Li et al., 2011; Zhou et al., 2018)
Dioscin (a spirostane glycoside)	Rhizome of *Dioscorea spongiosa*	Significantly reduces serum urate, anti-inflammatory, antiallergic	Downregulates Glut9 and upregulates Oat1	Rats with PO-induced hyperuricemia and mice with adenine-/PO-induced hyperuricemia	(Tao et al., 2018; Zhang et al., 2018)
RA-3 (a lanosteryl triterpene)	*Protorhus longifolia* stem bark	Significantly reduces serum urate, nephroprotective and antihyperglycemic	Inhibits XOD, downregulates pGSK-3β and pAKT	Sprague‒Dawley rat model of sepsis peritonitis, high-fat diet-fed and streptozotocin-induced T2DM rat model	Hlophe et al., (2020)
Theobromine	Cocoa	Markedly inhibits uric acid nucleation	—	—	Grases et al., (2014)
Caffeine	Coffee	Reduces serum urate	—	Systemic meta-analysis	(Bae et al., 2015; Park et al., 2016)
Curcumin	Rhizome of *Curcuma longa*	Significantly reduces serum urate, modulates the gut microbiota, fortifies the intestinal barrier, attenuates metabolic endotoxemia, protects renal function	—	Rats with PO-induced hyperuricemia	(Zhang et al., 2012; Xu et al., 2021)

These results suggest that TCM herbal extracts have potential benefits in the management of hyperuricemia and related kidney impairment. Due to the complex ingredient structures and compositions of TCM herbal extracts, it is difficult to comprehensively elucidate the mechanisms of the pharmacological effects of the extracts. Identification and exploration of natural bioactive compounds from TCMs might be promising directions for hyperuricemia management.

### The PI3K/Akt signaling pathway, a potential target for the treatment of hyperuricemia

Generally, MSU deposition causes acute gout flares and recurrent gout attacks that destroy joints. Macrophages are thought to initiate and drive MSU-induced inflammation. MSU-induced acute gouty arthritis in mice has been shown to cause activation of the PI3K/Akt pathway (Cao et al., 2021). In addition, insulin stimulation of urate uptake in human proximal tubular cells (PTC-05) and HEK293T cells is effectively abrogated by inhibitors of protein tyrosine kinase and PI3K (Mandal et al., 2021). In an oocyte expression system, insulin was shown to stimulate the urate transport activity of GLUT9a, GLUT9b, OAT10, OAT3, OAT1, NPT1, and ABCG2, which are directly activated by insulin signaling through the PI3K/Akt signaling pathway (Mandal et al., 2021). Anthocyanins, a group of natural flavonoids found in Korean black beans (also found in blue-, purple-, and red-colored fruits, flowers, and leaves) that regulate the PI3K/Akt/GSK3β pathways (Ali et al., 2018), have been reported to significantly reduce serum urate levels and XOD activity in the serum and livers of mice with yeast extract-induced hyperuricemia (Qian et al., 2019). Future studies are warranted to investigate whether anthocyanins regulate the expression of urate transporters *via* the PI3K/Akt signaling pathway. Chrysin, a flavonoid compound naturally found in honey, propolis and mushrooms that exerts anti-inflammatory and antioxidant effects, has been shown to effectively reduce serum urate levels by inhibiting the activity of XOD in the liver of high-fructose corn syrup-fed hyperuricemia rats by downregulating the protein expression of Urat1 and Glut9 and upregulating the protein expression of Oat1 and Abcg2 (Chang et al., 2021). The anti-inflammatory effect of chrysin is mediated by the PI3K/Akt/mTOR signaling pathway in RAW264.7 cells (Cai et al., 2021). Bergenin, a type of polyphenol compound with antiulcerogenic, anti-inflammatory, and wound-healing properties that induces ABCG2 expression and suppresses *SLC2A9* expression by inhibiting the nuclear translocation of p53 in HK-2 cells, reduces serum urate levels in mice with yeast polysaccharide-induced hyperuricemia by promoting renal and gut urate excretion (Chen et al., 2020).

### The JAK/STAT3 signaling pathway, a potential target for the treatment of hyperuricemia

Kidney fibrosis is a histologic hallmark of CKD that is possibly caused by hyperuricemia. Pharmacological inhibition of the activation of the JAK/STAT3 pathway has been shown to reduce serum urate levels and delay the progression of kidney fibrosis and CKD in adenine- and PO-induced hyperuricemia mouse models (Pan et al., 2021b). Berberrubine is an isoquinoline alkaloid that is the primary metabolite of berberine, the main component of Phellodendri Chinensis Cortex, which is found in *Coptis chinensis* Franch and *Phellodendron chinense* Schneid (Cheng H. et al., 2022). Berberrubine possesses antihyperuricemic and antigout effects and significantly decreases the serum urate levels in mice with PO- and hypoxanthine-induced hyperuricemia (Lin et al., 2021). Berberrubine has also been shown to reduce hepatic XOD activity, downregulate the expression of Glut9 and Urat1 and upregulate the expression of Oat1/3 and Abcg2 at both the protein and mRNA levels in mice with hyperuricemia as well as to suppress activation of the JAK/STAT3 signaling pathway (Lin et al., 2021). The natural flavonoid pectolinarigenin has been reported to significantly reduce serum urate levels in mice with adenine- and PO-induced hyperuricemic nephropathy (Ren et al., 2021a). Pectolinarigenin also inhibits the expression of transforming growth factor-beta (TGF-β)1 as well as the phosphorylation of the transcription factors Smad3 and Stat3, suggesting that suppression of inflammation and fibrosis by pectolinarigenin occurs through inhibition of Smad3 and Stat3 signaling pathway activation in mice with hyperuricemic nephropathy (Ren et al., 2021a). Fisetin (3,3′,4′,7-tetrahydroxyflavone), a naturally occurring flavonol, reduces serum urate by modulating the expression of kidney urate transporters, including Urat1, Oat1/3, and Abcg2, *via* the STAT3 and TGF-β signaling pathways in mice with PO- and adenine-induced hyperuricemia (Ren et al., 2021b). Fisetin treatment also reduces the levels of proinflammatory mediators, including tumor necrosis factor-alpha (TNF-α), interleukin 6 (IL-6) and monocyte chemoattractant protein-1; attenuates kidney fibrosis; and restores the expression of alpha-smooth muscle actin, collagen I and fibronectin *via* modulation of the STAT3 and TGF-β signaling pathways (Ren et al., 2021b). In a cell-based urate transport assay, fisetin was shown to be a strong URAT1 inhibitor with a half-maximal inhibitory concentration of 7.5 µM (Toyoda et al., 2022). Curcumin has been shown to reduce fructose-induced hyperuricemia and renal endothelial dysfunction by inhibiting activation of NO-mediated JAK/STAT signaling in rats (Zhang et al., 2012).

### NLRP3 and the NF-κB/TLR4 signaling pathway, potential targets for the treatment of hyperuricemia

The inflammasome is a by multiprotein cytoplasmic complex that plays important roles in host defense and inflammatory responses through activating caspase-1 and promoting secretion of the proinflammatory cytokines interleukin 1beta (IL-1β) and IL-18 (Franchi et al., 2009). Nod-like receptor protein members, including NLRP1, NLRP3 and NLRC4, and the adaptor ASC (apoptosis-related specific protein) constitute the inflammasome (Franchi et al., 2009). The NLRP3 inflammasome, an intracellular sensor, plays an important role in innate immunity and is therefore the most investigated inflammasome (Franchi et al., 2009). For activation of the NLRP3 inflammasome, activation of NF-κB is required to upregulate the expression of NLRP3, pro-IL-1β, and caspase 1, which is accomplished *via* stimulation of Toll-like receptors (TLRs) (Toma et al., 2010; Qiao et al., 2012). MSU, identified as a danger signal formed after the release of uric acid from dying cells (Shi et al., 2003), has been shown to trigger the cellular inflammatory response through the NLRP3 inflammasome, resulting in the production of the active inflammatory cytokines IL-1β and IL-18 (Martinon et al., 2006). MSU-induced inflammation and oxidative stress proceed through the NF-κB/NLRP3 and Nrf2 pathways, leading to increased production of the inflammatory cytokines IL-1β, IL-6, IL-18, and TNF-α (Alberts et al., 2019; Cheng J.-J. et al., 2022). The bacterial cell wall component crude LPS can also activate the NRLP3 inflammasome (Martinon et al., 2006).

In this review, we have shown the mechanisms by which some natural bioactive compounds suppress activation of the NLRP3 inflammasome in mice with hyperuricemia (Table 1). In a PO-induced hyperuricemia mouse model, reduction of serum urate by treatment with the XOD inhibitor N-(9,10-anthraquinone-2-yl-carbonyl), downregulates Glut9 protein expression and upregulates Oat1 and Oat3 protein expression, resulting in decrease in the levels of TNF-α, IL-6, and other inflammatory factors in the serum and kidney of mice and inhibition of NLRP3 pathway-mediated inflammation (Gao et al., 2022). Chlorogenic acid, a polyphenolic compound found in medicinal herbs with antioxidant and anti-inflammatory activity, has been shown to inhibit XOD and downregulate the mRNA expression of secretory uric acid transporters in a hypoxanthine- and PO-induced hyperuricemia mouse model (Zhou et al., 2021). In addition, chlorogenic acid reduces the mRNA expression of IL-1β, TNF-α, Nlrp3, and caspase-1. It also inhibits activation of the TLR 4/MyD88/NF-κB signaling pathway in the kidney and reduces the mRNA expression of ileal IL-1β and IL-6, resulting in inflammation relief in the above hyperuricemic mice and in LPS-induced acute kidney injury (Ye et al., 2017; Zhou et al., 2021). Berberrubine has been shown to significantly reduce serum urate levels and the levels of inflammatory mediators (IL-1β, IL-6, and TNF-α) in mice with PO- and hypoxanthine-induced hyperuricemia (Lin et al., 2021). Pectolinarigenin, in addition to reducing serum urate in mice with hyperuricemic nephropathy (Ren et al., 2021a), inhibits LPS-induced NF-κB activation by interfering with the degradation of IκB-α and the synthesis of inducible nitric oxide synthase, cyclooxygenase-2, IL-6, IL-1β, and TNF-α in RAW264.7 and THP1 cell lines (Feng et al., 2022). Chrysin improves vascular permeability and alleviates the inflammatory response in lung tissue by suppressing the IRE1α/TXNIP/NLRP3 signaling pathway, thereby alleviating LPS-induced acute lung injury in mice (Chen M. et al., 2021). Bergenin reduced the levels of serum urate, IL-6, IL-1β, and TNF-α in hyperuricemic mice and promotes a polarization shift from the M1 to the M2 phenotype in RAW264.7 cells (Chen et al., 2020). Esculetin is a natural dihydroxy coumarin found in *Fraxinus rhynchophylla* bark and chicory skin that has been shown to reduce serum urate by increasing renal urate excretion, inhibiting XOD expression and activity in the liver, and modulating urate transporters in the kidney (Hong et al., 2014; Wang et al., 2020). Esculetin has been shown to attenuate elevations in the levels of proinflammatory cytokines, including IL-6, IL-1β, and TNF-α, and serum urate and suppress inducible nitric oxide synthase and cyclooxygenase-2 protein expression by blocking the NF-κB pathway and suppressing the generation of proinflammatory mediators, including nitric oxide and prostaglandin E2, in LPS-induced RAW264.7 macrophages and mice (Hong et al., 2014; Zhu et al., 2016). Huang et al. reported that fisetin, a naturally occurring flavonol, inhibits inflammatory responses in experimental periodontitis in rats and LPS-induced human gingival fibroblasts through the FGFR1/TLR4/NLRP3 inflammasome pathway, which mitigates kidney injury to restore the normal expression of Urat1 (Huang et al., 2021). Phloretin, a dihydrochalcone, is a type of natural phenol found in the leaves of apple and apricot trees with antioxidant and anti-inflammatory properties. Phloretin has been shown to effectively attenuate urate-induced renal injury by inhibiting Nlrp3 and urate reabsorption mediated by Glut9 and promoting urinary urate excretion in mice with PO-/adenine-induced hyperuricemia (Cui et al., 2020). In human umbilical vein endothelial cells, phloretin also significantly attenuates proinflammatory factor expression and reduces GLUT9-mediated urate uptake by inhibiting activation of the ERK/NF-κB pathway (Liu et al., 2017). Resveratrol, a polyphenolic, non-flavonoid plant-derived antitoxin, has been shown to reduce serum urate levels by downregulating the expression levels of Glut9 and Urat1 and to improve kidney inflammation, possibly *via* the TLR4 AND NLRP3 signaling pathways, in mice with high-fat diet-induced insulin resistance (Zhang et al., 2021). In a classic Sprague‒Dawley rat model of sepsis peritonitis, resveratrol significantly decreases LPS-induced expression of NF-κB, TNF-α, IL6, IL-1β, and TLR4 and increases the expression of p-PI3K, p-AKT, and p-mTOR in the myocardium (Shang et al., 2019). Thus, resveratrol has the potential to protect the myocardium in sepsis by activating the PI3K/AKT/mTOR signaling pathway and inhibiting the NF-κB signaling pathway and related inflammatory factors (Shang et al., 2019). Resveratrol also reduces LPS-induced inflammation by reducing the levels of inflammatory cytokines (IL-6, IL-18 and TNF-α) in duck ileitis (Yang et al., 2021). Bergenin, a C-glucoside of 4-O-methyl gallic acid isolated from several medicinal plants, has been shown to reduce serum urate levels in a hyperuricemia-induced mouse model by elevating Abcg2 expression in both the kidney and intestine and by suppressing *Slc2a9* expression in the kidney (Chen et al., 2020). Bergenin also reduces the serum levels of IL-6, IL-1β, and TNF-α in hyperuricemic mice (Chen et al., 2020). Baicalein, a bioactive flavonoid exhibiting antioxidant, anti-inflammatory, antihypertensive and anticancer properties, has been reported to significantly reduce serum urate and enhance renal urate excretion in mice with PO-induced hyperuricemia by specifically inhibiting the [^14^C]-urate uptake activities of Glut9 and Urat1 in a noncompetitive manner, with a half-maximal inhibitory concentration values of 30.17 ± 8.68 and 31.56 ± 1.37 μM, respectively (Chen Y. et al., 2021). Baicalein also downregulates Glut9 and Urat1 expression in the kidney and suppressed XOD activity in the serum and liver (Chen Y. et al., 2021). Cisplatin resistance is one of the major obstacles in the treatment of non-small cell lung cancer. Combining baicalein with cisplatin has been shown to significantly attenuate cisplatin resistance in human A549 lung adenocarcinoma cells through inhibition of the PI3K/AKT/NF-κB pathway (Yu et al., 2017).

## Present-day first-line medications for the management of hyperuricemia/gout

It is not clear whether elevated uric acid is the primary cause of kidney disease or the oxidant (H_2_O_2_) produced as byproduct by the activation of XOD in addition to uric acid. Are uric acid-lowering agents more beneficial than angiotensin-converting enzyme inhibitors in subjects with CKD? At present, the first-line medications for hyperuricemia/gout are XOD inhibitors, such as allopurinol (a purine analog and competitive xanthine oxidase inhibitor), or the more renoprotective febuxostat/topiroxostat (a nonpurine analog), which inhibits uric acid production (Figure 1). Although allopurinol can successfully control serum urate levels in hyperuricemia/gout patients, long-term use of a high dose of allopurinol to promote the dissolution of urate crystals has been linked to an increased risk of liver, kidney and cardiovascular diseases (Arellano and Sacristán, J. A. 1993; Yang et al., 2015). Febuxostat is used only in the subset of CKD patients with impaired renal function due to its hepatic elimination. There is a risk that all xanthine oxidase inhibitors can increase urinary xanthine levels, which can be nephrotoxic. Uricosurics lower uric acid levels by promoting urate excretion through interactions with urate transporters. Uricosuric diuretics such as probenecid and benzbromarone increase urine output, but high-dose or long-term use of diuretics increases the risk of hepatotoxicity and renal failure (Stamp and Chapman, 2014). Probenecid is ineffective in patients with renal impairment (Stamp et al., 2005). Benzbromarone is effective in allopurinol-intolerant patients with renal failure, solid organ transplant or tophaceous/polyarticular gout. In an oocyte expression system, benzbromarone has been found to inhibit ABCG2, the major transporter for urate excretion in the kidney and intestine, along with OAT1, OAT3 and NPT1 (Mandal et al., 2017). Notably, benzbromarone was withdrawn from the market by Sanofi-Synthelabo in 2003, after reports of serious hepatotoxicity. Fenofibrate may reduce serum urate but it is associated with the decline in renal function (Stamp et al., 2005). In transplant recipients, there is a risk of adverse effects and potentially severe interactions between hypouricemic and immunosuppressive drugs (Stamp et al., 2005).

## Conclusion

Hyperuricemia is associated with multiple comorbidities, including metabolic syndrome, CKD, urolithiasis, cardiovascular disease, hypertension, dyslipidemia and diabetes, creating a demand for new antihyperuricemic and nephroprotective drugs. Long-term use of high doses of first-line medications for hyperuricemia likely induces acute kidney disease, CKD, liver toxicity, and adverse effects in transplant recipients. Therefore, there is an urgent need for new nephroprotective drugs with improved safety profiles and tolerance. To avoid oxidative stress and excessive inflammatory responses, new therapeutic drugs with strong antioxidant and anti-inflammatory activities would be preferred. There is substantial evidence of naturally occurring bioactive compounds from various medicinal plants with antihyperuricemic and nephroprotective potential that can protect the kidney from various insults and maintain their integrity and functions. The current review has covered some of the compounds that influence the expression and activity of urate transporters, including URAT1, GLUT9, ABCG2, NPTs, and OATs, and their regulatory pathways, such as the PI3K/AKT, JAK/STAT3, NLRP3, and NF-κB pathways.

## Abbreviations

ABCG2, ATP-binding cassette transporter, subfamily G, member 2; CKD, chronic kidney disease; GLUT9, glucose transporter member 9; IL-1β, interleukin-1β; IL-6, interleukin-6; JAK/STAT3, Janus kinase/signal transducer and activator of transcription 3, MSU, monosodium urate; NF-κB, nuclear factor-kappa B; NLRP3, NOD-like receptor family pyrin domain-containing 3; NPT, sodium-dependent phosphate cotransporter type; OAT, organic anion transporter; PI3K/AKT, phosphoinositide 3-kinase/protein kinase; PO, potassium oxonate; ROS, reactive oxygen species; LPS, lipopolysaccharide; SNPs, single nucleotide polymorphisms; TCMs, traditional Chinese medicines, TGF-β, transforming growth factor-beta; TNF-α, tumor necrosis factor-alpha; TLR4, Toll-like receptor 4; URAT1, human urate transporter 1; XOD, xanthine oxidoreductase.
